# Supplementary material for: Exploring Literacy and Knowledge Gaps and Disparities in Genetics and Oncogenomics Among Cancer Patients and the General Population: A Scoping Review
Source: Healthcare (Basel). 2025 Jan 9;13(2):121. doi: 10.3390/healthcare13020121 (PMC11765264; doi:10.3390/healthcare13020121)
Supplement: Supplementary file 1 [file healthcare-13-00121-s001.zip › healthcare-3350337-supplementary.pdf]

Supplementary tables

Table S1. Search strategy - PubMed

Search details
(("genetic counseling"[Title/Abstract] OR "genetic testing"[Title/Abstract] OR "personalised medicine"[Title/Abstract] OR "oncogenom*"[Title/Abstract] OR "genom*"[Title/Abstract] OR "next generation sequencing"[Title/Abstract] OR "genetic*"[Title/Abstract]) AND ("cancer predisposition"[Title/Abstract] OR "hereditary cancer syndromes"[Title/Abstract] OR "malignancy"[Title/Abstract] OR "neoplasm*"[Title/Abstract] OR "oncology"[Title/Abstract] OR "cancer"[Title/Abstract]) AND ("pediatric*"[Title/Abstract] OR "caregiver*"[Title/Abstract] OR "student*"[Title/Abstract] OR "adolescent*"[Title/Abstract] OR "youth"[Title/Abstract] OR "general public"[Title/Abstract] OR "citizen*"[Title/Abstract] OR "patient*"[Title/Abstract]) AND ("understanding"[Title/Abstract] OR "awareness"[Title/Abstract] OR "literacy"[Title/Abstract] OR "information needs"[Title/Abstract] OR "knowledge"[Title/Abstract])) AND (2018/1/1:2023/1/2[pdat])
("genetic counseling"[Title/Abstract] OR "genetic testing"[Title/Abstract] OR "personalised medicine"[Title/Abstract] OR "oncogenom*"[Title/Abstract] OR "genom*"[Title/Abstract] OR "next generation sequencing"[Title/Abstract] OR "genetic*"[Title/Abstract]) AND ("cancer predisposition"[Title/Abstract] OR "hereditary cancer syndromes"[Title/Abstract] OR "malignancy"[Title/Abstract] OR "neoplasm*"[Title/Abstract] OR "oncology"[Title/Abstract] OR "cancer"[Title/Abstract]) AND ("pediatric*"[Title/Abstract] OR "caregiver*"[Title/Abstract] OR "student*"[Title/Abstract] OR "adolescent*"[Title/Abstract] OR "youth"[Title/Abstract] OR "general public"[Title/Abstract] OR "citizen*"[Title/Abstract] OR "patient*"[Title/Abstract]) AND ("understanding"[Title/Abstract] OR "awareness"[Title/Abstract] OR "literacy"[Title/Abstract] OR "information needs"[Title/Abstract] OR "knowledge"[Title/Abstract])
"genetic counseling"[Title/Abstract] OR "genetic testing"[Title/Abstract] OR "personalised medicine"[Title/Abstract] OR "oncogenom*"[Title/Abstract] OR "genom*"[Title/Abstract] OR "next generation sequencing"[Title/Abstract] OR "genetic*"[Title/Abstract]
"genetic counseling"[Title/Abstract]
"genetic testing"[Title/Abstract]
"personalised medicine"[Title/Abstract]
"oncogenom*"[Title/Abstract]
"genom*"[Title/Abstract]
"next generation sequencing"[Title/Abstract]
"genetic*"[Title/Abstract]
"cancer predisposition"[Title/Abstract] OR "hereditary cancer syndromes"[Title/Abstract] OR "malignancy"[Title/Abstract] OR "neoplasm*"[Title/Abstract] OR "oncology"[Title/Abstract] OR "cancer"[Title/Abstract]
"cancer predisposition"[Title/Abstract]
"hereditary cancer syndromes"[Title/Abstract]
"malignancy"[Title/Abstract]
"neoplasm*"[Title/Abstract]
"oncology"[Title/Abstract]
"cancer"[Title/Abstract]
"pediatric*"[Title/Abstract] OR "caregiver*"[Title/Abstract] OR "student*"[Title/Abstract] OR "adolescent*"[Title/Abstract] OR "youth"[Title/Abstract] OR "general public"[Title/Abstract] OR "citizen*"[Title/Abstract] OR "patient*"[Title/Abstract]
"pediatric*"[Title/Abstract]
"caregiver*"[Title/Abstract]
"student*"[Title/Abstract]
"adolescent*"[Title/Abstract]
"youth"[Title/Abstract]
"general public"[Title/Abstract]
"citizen*"[Title/Abstract]
"patient*"[Title/Abstract]
"understanding"[Title/Abstract] OR "awareness"[Title/Abstract] OR "literacy"[Title/Abstract] OR "information needs"[Title/Abstract] OR "knowledge"[Title/Abstract]
"understanding"[Title/Abstract]
"awareness"[Title/Abstract]
"literacy"[Title/Abstract]
"information needs"[Title/Abstract]
"knowledge"[Title/Abstract]

Table S2. Search strategy - Scopus

((TITLE-ABS (cancer) OR TITLE-ABS (oncology) OR TITLE-ABS (neoplasms) OR TITLE-ABS (malignancy) OR TITLE-ABS (hereditary AND cancer AND syndromes) OR TITLE-ABS (cancer AND predisposition))) AND ((TITLE-ABS (patient*) OR TITLE-ABS (citizen*) OR TITLE-ABS (general AND public) OR TITLE-ABS (youth) OR TITLE-ABS (adolescent*) OR TITLE-ABS (student*) OR TITLE-ABS (caregiver*) OR TITLE-ABS (pediatric*))) AND ((TITLE-ABS (genetic*) OR TITLE-ABS (genome) OR TITLE-ABS (genomic) OR TITLE-ABS (oncogenomic*) OR TITLE-ABS (personalised PRE/0 medicine) OR TITLE-ABS (gene) OR TITLE-ABS (genetic PRE/1 testing) OR TITLE-ABS (genetic PRE/0 counseling) OR TITLE-ABS (next PRE/0 generation PRE/0 sequencing))) AND ((TITLE-ABS (knowledge) OR TITLE-ABS (understanding) OR TITLE-ABS (information W/3 needs) OR TITLE-ABS (literacy) OR TITLE-ABS (awareness))) AND PUBYEAR > 2017 AND PUBYEAR < 2024 AND (LIMIT-TO (DOCTYPE , "ar"))
((TITLE-ABS-KEY (cancer) OR TITLE-ABS-KEY (oncology) OR TITLE-ABS-KEY (neoplasms) OR TITLE-ABS-KEY (malignancy) OR TITLE-ABS-KEY (hereditary AND cancer AND syndromes) OR TITLE-ABS-KEY (cancer AND predisposition))) AND ((TITLE-ABS-KEY (patient*) OR TITLE-ABS-KEY (citizen*) OR TITLE-ABS-KEY (general AND public) OR TITLE-ABS-KEY (youth) OR TITLE-ABS-KEY (adolescent*) OR TITLE-ABS-KEY (student*) OR TITLE-ABS-KEY (caregiver*) OR TITLE-ABS-KEY (pediatric*))) AND ((TITLE-ABS-KEY (genetic*) OR TITLE-ABS-KEY (genome) OR TITLE-ABS-KEY (genomic) OR TITLE-ABS-KEY (oncogenomic*) OR TITLE-ABS-KEY (personalised PRE/0 medicine) OR TITLE-ABS-KEY (gene) OR TITLE-ABS-KEY (genetic PRE/1 testing) OR TITLE-ABS-KEY (genetic PRE/0 counseling) OR TITLE-ABS-KEY (next PRE/0 generation PRE/0 sequencing))) AND ((TITLE-ABS-KEY (knowledge) OR TITLE-ABS-KEY (understanding) OR TITLE-ABS-KEY (information W/3 needs) OR TITLE-ABS-KEY (literacy) OR TITLE-ABS-KEY (awareness))) AND PUBYEAR > 2017 AND PUBYEAR < 2024 AND (LIMIT-TO (DOCTYPE , "ar"))
((TITLE-ABS-KEY (cancer) OR TITLE-ABS-KEY (oncology) OR TITLE-ABS-KEY (neoplasms) OR TITLE-ABS-KEY (malignancy) OR TITLE-ABS-KEY (hereditary AND cancer AND syndromes) OR TITLE-ABS-KEY (cancer AND predisposition))) AND ((TITLE-ABS-KEY (patient*) OR TITLE-ABS-KEY (citizen*) OR TITLE-ABS-KEY (general AND public) OR TITLE-ABS-KEY (youth) OR TITLE-ABS-KEY (adolescent*) OR TITLE-ABS-KEY (student*) OR TITLE-ABS-KEY (caregiver*) OR TITLE-ABS-KEY (pediatric*))) AND ((TITLE-ABS-KEY (genetic*) OR TITLE-ABS-KEY (genome) OR TITLE-ABS-KEY (genomic) OR TITLE-ABS-KEY (oncogenomic*) OR TITLE-ABS-KEY (personalised PRE/0 medicine) OR TITLE-ABS-KEY (gene) OR TITLE-ABS-KEY (genetic PRE/1 testing) OR TITLE-ABS-KEY (genetic PRE/0 counseling) OR TITLE-ABS-KEY (next PRE/0 generation PRE/0 sequencing))) AND ((TITLE-ABS-KEY (knowledge) OR TITLE-ABS-KEY (understanding) OR TITLE-ABS-KEY (information W/3 needs) OR TITLE-ABS-KEY (literacy) OR TITLE-ABS-KEY (awareness))) AND PUBYEAR > 2017 AND PUBYEAR < 2024
((TITLE-ABS-KEY (cancer) OR TITLE-ABS-KEY (oncology) OR TITLE-ABS-KEY (neoplasms) OR TITLE-ABS-KEY (malignancy) OR TITLE-ABS-KEY (hereditary AND cancer AND syndromes) OR TITLE-ABS-KEY (cancer AND predisposition))) AND ((TITLE-ABS-KEY (patient*) OR TITLE-ABS-KEY (citizen*) OR TITLE-ABS-KEY (general AND public) OR TITLE-ABS-KEY (youth) OR TITLE-ABS-KEY (adolescent*) OR TITLE-ABS-KEY (student*) OR TITLE-ABS-KEY (caregiver*) OR TITLE-ABS-KEY (pediatric*))) AND ((TITLE-ABS-KEY (genetic*) OR TITLE-ABS-KEY (genome) OR TITLE-ABS-KEY (genomic) OR TITLE-ABS-KEY (oncogenomic*) OR TITLE-ABS-KEY ("personalised medicine") OR TITLE-ABS-KEY (gene) OR TITLE-ABS-KEY ("genetic testing") OR TITLE-ABS-KEY ("genetic counseling") OR TITLE-ABS-KEY ("next generation sequencing"))) AND ((TITLE-ABS-KEY (knowledge) OR TITLE-ABS-KEY (understanding) OR TITLE-ABS-KEY ("information needs") OR TITLE-ABS-KEY (literacy) OR TITLE-ABS-KEY (awareness)))
(TITLE-ABS-KEY (knowledge) OR TITLE-ABS-KEY (understanding) OR TITLE-ABS-KEY ("information needs") OR TITLE-ABS-KEY (literacy) OR TITLE-ABS-KEY (awareness))
(TITLE-ABS-KEY (genetic*) OR TITLE-ABS-KEY (genome) OR TITLE-ABS-KEY (genomic) OR TITLE-ABS-KEY (oncogenomic*) OR TITLE-ABS-KEY ("personalised medicine") OR TITLE-ABS-KEY (gene) OR TITLE-ABS-KEY ("genetic testing") OR TITLE-ABS-KEY ("genetic counseling") OR TITLE-ABS-KEY ("next generation sequencing"))
(TITLE-ABS-KEY (patient*) OR TITLE-ABS-KEY (citizen*) OR TITLE-ABS-KEY (general AND public) OR TITLE-ABS-KEY (youth) OR TITLE-ABS-KEY (adolescent*) OR TITLE-ABS-KEY (student*) OR TITLE-ABS-KEY (caregiver*) OR TITLE-ABS-KEY (pediatric*))
(TITLE-ABS-KEY (cancer) OR TITLE-ABS-KEY (oncology) OR TITLE-ABS-KEY (neoplasms) OR TITLE-ABS-KEY (malignancy) OR TITLE-ABS-KEY (hereditary AND cancer AND syndromes) OR TITLE-ABS-KEY (cancer AND predisposition))

Table S3. Characteristics of the selected studies (n=43)

Author, Year	Country, City (or State)	Time frame	Study type	Knowledge assessment tool	Population	N	Age	G/GT* Status
**Aizuddin et al., 2021 (13)**	Malaysia, Kuala Lumpur	Jan-Feb 2017	Cross-sectional study	Ten-item self-administered questionnaire	All types of cancer patients, along with their caregivers, and citizens	175	49.29 (SD 15.2)	4.6% had undergone G/GT
**Oberg et al., 2018 (53)**	USA, New York	Aug 2015-Jun 2016	Cross-sectional study	Precision in Pediatric Sequencing Knowledge Questionnaire (PIPseqKQ)	Young adult cancer survivors and their parents	111	≥ 18	Not offered G/GT
**Butow et al., 2022 (14)**	Australia, Sydney	Jan 2020-Aug 2021	Cohort study	Knowledge of genome sequencing questionnaire (KOGS)	Individuals with a personal history of cancer	261	41.4 (SD 14.2)	About to undergo G/GT
**Johnson et al., 2019 (42)**	USA, New York	Aug 2015-Aug 2017	Clinical trial	Genetics Knowledge Quiz (GKQ)	Parents of children with cancer	158	37.4 (SD 7.7)	Their children were about to undergo NGS
**Makhnoon et al., 2021 (15)**	USA, Washington	2016	Clinical trial	11-item validated genetic knowledge measure	Active cancer patients	189	67.4 (32-103)	About to undergo GS or panel test
**Wing et al., 2021 (16)**	USA, California	2018-2019	Cross-sectional study	Questionnaire adapted from Blanchette et al.	Cancer patients	85	30-89	Had undergone G/GT and had received results
**Hamilton et al., 2019 (38)**	USA, New York	Sep-Dec 2017	Cohort study	Modified University of North Carolina Genomic Knowledge Scale	Adults tested for cancer susceptibility genes	57	50.9 6 (SD 10.8)	Had undergone MGPT and had received results
**Anderson et al., 2021 (17)**	USA, Maine	Jul 2017-Oct 2020	Clinical trial	Four-item questionnaire based on previous research	Cancer patients	1139	64 (SD 11)	Offered large-panel genomic tumor testing
**Roth et al., 2021 (18)**	USA, Washington	Aug 2017-Jun 2019	Clinical trial	Four-item questionnaire	Patients with advanced non-small-cell lung cancer	207	66.8 (SD 8.1)	About to undergo genomic testing.
**Puryear et al., 2017 (45)**	USA, California	May-Sep 2015	Mixed methods	Tool adapted from the Genetic Literacy and Comprehension Measure	Primary care adult patients	97	54.8	Not offered NGS
**Bon et al., 2022 (43)**	Netherlands, Utrecht	Feb 2019-Jul 2021	Cohort study	Semi-structured Interviews	Parents of children with cancer	29	≥ 18	Their children had undergone NGS and half of them had received results
**Xiao et al., 2020 (44)**	China, Guangzhou	Dec 2017-Dec 2018	Cross-sectional study	7-item questionnaire	Parents of children with cancer	126	≥ 18	38/126 children had undergone G/GT
**Roberts et al., 2019 (19)**	USA, Michigan	Apr 2014-Dec 2016	Clinical trial	General knowledge of genome sequencing scale	Adult patients with advanced-stage solid tumor malignancies	217	59.1 (SD 12.0)	About to undergo G/GT
**Adams et al., 2020 (20)**	USA, Ohio	Sep 2013-Oct 2015	Clinical trial	Subjective and objective genetic knowledge scale	Patients with metastatic breast cancer	58	≥ 18	About to undergo somatic genomic testing. Half of them had previously undergone G/GT

**Gómez-Trillos et al., 2020 (36)**	USA, Washington	2019	Qualitative	Interviews	Hispanic/Latinas meeting 2015 NCCN guidelines for breast and/or ovarian cancer genetic assessment	20	44.86 (SD 6.61)	8 participants reported having received genetic testing and/or counseling
**Mullally et al., 2021 (21)**	Ireland, Dublin	Nov 2018	Cross-sectional study	14-item questionnaire	Active cancer patients	84	56 (26–83)	Not offered G/GT
**Hill et al., 2018 (54)**	Canada, Toronto	Oct-Dec 2015	Qualitative	Interviews	Adult retinoblastoma survivors and parents of children with retinoblastoma	15	≥ 18	Most participants had undergone G/GT
**Guo et al., 2022 (46)**	USA, Texas	May-Jul 2017	Cross-sectional study	9-item questionnaire	Women from low-income families	677	18–65	Not offered G/GT
**Gornick et al., 2018 (22)**	USA, California	Feb 2014-May 2016	Clinical trial	Tool designed for the study	Newly diagnosed patients with breast cancer	496	≥ 18	95.41% had undergone G/GT
**Pozzar et al. 2022 (23)**	USA, Massachusetts	Jun-Aug 2017	Cross-sectional study	KnowGene scale	Adults with a personal history of breast or gynecologic cancer	87	65.2 (SD 10.5)	Had undergone genetic counselling and testing
**Shin et al., 2021 (24)**	Korea, Goyang	Jun-Oct 2018	Cross-sectional study	12-item questionnaire	Patients newly diagnosed with epithelial ovarian, fallopian, or primary peritoneal cancer	103	53.11 (SD 11.5)	95.2% had undergone genetic testing using NGS
**Underhill-Blazey et al., 2021 (34)**	USA, Massachusetts	Jun-Aug 2017	Mixed methods	KnowGene scale	Adults with a breast or gynecologic cancer diagnosis	602	58 (SD 24-91)	Had undergone MGPT
**Saya et al., 2022 (47)**	Australia, Melbourne	Feb-Nov 2018	Cohort study	Multidimensional Measure of Informed Choice (MMIC)	Adults who were offered a genomic CRC risk test	150	45–74	Were offered a genomic test for CRC risk
**Park et al., 2022 (35)**	Korea, Seoul	Mar 2016-Dec 2019	Cross-sectional study	Tool adapted from Erblich's Breast Cancer Genetic Counseling Knowledge Questionnaire (BGKQ)	BRCA1/2 mutation-negative breast cancer patients with at least one high-risk factor for hereditary breast cancer syndrome	347	18-83	Had undergone MGPT and genetic counseling
**Marron et al., 2019 (33)**	USA, Massachusetts	Sep 2012-Oct 2013	Clinical Trial	Genetic Knowledge Index (GKI)	Children cancer patients and caregivers	45		The children had undergone NGS
**Underhill-Blazey et al., 2019 (25)**	USA, Massachusetts	Jan 2016-Oct 2017	Cross-sectional study	KnowGene scale	Cancer patients with breast or gynaecological cancer	591	58 (24–91)	Had undergone multigene panel testing
**Dehar et al., 2022 (26)**	Canada, Alberta	Jul-Sep 2019	Cross-sectional study	Tool developed for the study	Cancer patients	113	63 (33–93)	Had received genetic testing
**McCuaig et al., 2021 (27)**	Canada, Toronto	Jan 2017-Aug 2019	Clinical Trial	A novel 11-item knowledge tool	Breast and ovarian cancer patients	120	≥ 18	Were about to receive multi-gene panel genetic testing genetic testing
**Bartley et al., 2020 (28)**	Australia, Sydney	Aug 2017-May 2018	Clinical Trial	Semi-structured telephone interviews	Patients with cancer of possibly genetic aetiology	20	46 (32–78)	Had undergone GS
**Napier et al., 2022 (29)**	Australia, Sydney	Aug 2016-Aug 2019	Clinical Trial	Seven-item study-specific knowledge scale	Patients with cancer of possibly genetic aetiology	561	41.82 (13.72)	Had undergone GS
**Liang et al., 2018 (30)**	USA, California	Aug 2015-Jan 2016	Cross-sectional study	11-item tool	Ovarian cancer patients	53	57 (34-87)	64% of participants reported either having seen a genetic counselor or having

								undergone genetic testing
**Frost et al., 2019 (37)**	USA, Canada, Australia	2013-2014	Cohort study	Interviews	Cancer patients +family	32	52 (30-65)	54% had undergone genetic testing
**Krakow et al., 2018 (48)**	USA	Jan-Ap 2017	Cross-sectional study	Survey	A representative sample of civilian, non-institutionalised adults	1878	≥ 18 years	20.95% had undergone genetic testing of any kind
**Davies et al., 2020 (31)**	Australia, Sydney	2016-2019	Clinical Trial	An 8-item, multiple-choice, study-specific scale	Adult patients with pathologically confirmed advanced or metastatic solid cancers	777	55.47 (SD 14.3)	Were about to undergo MTP
**Best et al., 2019 (32)**	Australia, Sydney	Oct 2016-Jul 2019	Clinical Trial	Semi-structured interview	Adult participants with pathologically confirmed advanced or metastatic solid cancer, with a particular focus on rare cancer	20	57.1 (41–77)	Were about to undergo MTP
**Metcalfe et al., 2018 (49)**	Australia, Sydney, Melbourne	Jul–Sep 2015	Qualitative	Focus groups	Non-expert members of the public	56	18-80	7 had prior genetic testing and 10 were unsure
**Horrow et al., 2019 (50)**	USA, Rochester	2018-2019	Clinical research study	ClinSeq knowledge	Participants of a clinical study who were offered genomic testing	2895	60.1 (SD 8.06)	Were about to undergo genomic testing
**Alvord et al., 2020 (51)**	USA, Oregon	Oct 2017	Mixed methods	2-item Likert scale	Residents of Oregon	203	45.6 (SD 20.6)	Unknown
**Fogleman et al., 2019 (52)**	USA, Illinois	Oct 2015-Feb 2016	Cross-sectional study	2-item Likert scale	Residents of isolated communities (towns of roughly 1500 residents that were at least 20 miles from a town with a population greater than 5000)	114	≥ 18	Had undergone G/GT
**Robles-Rodriguez et al., 2024 (39)**	USA, New Jersey	2022	Qualitative	Focus groups	Women who had completed breast cancer treatment or they were living with metastatic cancer	29	55 (32-79)	Some had undergone G/GT
**Pramanik et al., 2024 (41)**	New Delhi, India	Jan-Dec 2018	Cross-sectional study	An in-house expert-validated questionnaire	Patients with a diagnosis of breast or ovarian cancer attending clinics for treatment or follow-up	84	25-75	Had not undergone G/GT
**Stallings et al., 2023 (55)**	USA, Tennessee	Jan-Jun 2019	Mixed-methods cross-sectional study	Survey and focus groups	Individuals at least 40 years of age with a personal experience with cancer (diagnosed with cancer, caregiver of someone diagnosed with cancer, and/or had a family member diagnosed with cancer)	26	60 (51–74)	4 had offered a GT
**Wang et al., 2023 (40)**	USA, California	2015	Cross-sectional study	A seven-item knowledge questionnaire	Adults who had been diagnosed with breast cancer within two years from the time of enrollment	93	Chinese Americans: 63.92 (SD 11.02) Non-Hispanic White: 57.4 (SD 12.35)	32 had undergone G/GT

* G/GT: Genetic or Genomic Testing
